# Bis[μ-4-(methyl­amino)­benzoato]-κ^3^ *O*,*O*′:*O*;κ^3^ *O*:*O*,*O*′-bis­{aqua­[4-(methyl­amino)­benzoato-κ^2^ *O*,*O*′](nicotinamide-κ*N*)cadmium(II)}

**DOI:** 10.1107/S1600536810046258

**Published:** 2010-11-13

**Authors:** Tuncer Hökelek, Ertuğrul Gazi Sağlam, Barış Tercan, Özgür Aybirdi, Hacali Necefoğlu

**Affiliations:** a Department of Physics, Hacettepe University, 06800 Beytepe, Ankara, Turkey; b Department of Chemistry, Ankara University, 06100 Tandoğan, Ankara, Turkey; c Department of Physics, Karabük University, 78050 Karabük, Turkey; d Department of Chemistry, Kafkas University, 36100 Kars, Turkey

## Abstract

In the dinuclear centrosymmetric Cd^II^ compound, [Cd_2_(C_8_H_8_NO_2_)_4_(C_6_H_6_N_2_O)_2_(H_2_O)_2_], the metal atom is chelated by two carboxyl­ate groups from 4-(methyl­amino)­benzoate (PMAB) anions, and coordinated by one nicotinamide and one water mol­ecule; a carboxyl­ate O atom from the adjacent PMAB anion bridges to the Cd atom, completing the irregular seven-coordination geometry. In the crystal, inter­molecular O—H⋯O, N—H⋯O and C—H⋯O hydrogen bonds link the mol­ecules into a three-dimensional network. π–π contacts between the pyridine rings [centroid–centroid distance = 3.965 (1) Å] may further stabilize the structure. A weak C—H⋯π inter­action also occurs.

## Related literature

For niacin, see: Krishnamachari (1974 ▶). For *N*,*N*-diethyl­nicotinamide, see: Bigoli *et al.* (1972 ▶). For related structures, see: Greenaway *et al.* (1984 ▶); Hökelek & Necefoğlu (1996 ▶); Hökelek *et al.* (2009*a* ▶,*b* ▶,*c* ▶,*d* ▶, 2010 ▶).
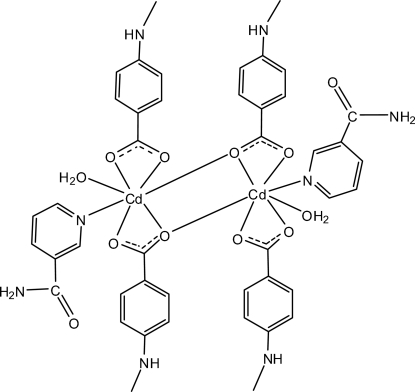


## Experimental

### 

#### Crystal data

[Cd_2_(C_8_H_8_NO_2_)_4_(C_6_H_6_N_2_O)_2_(H_2_O)_2_]
*M* *_r_* = 1105.72
Triclinic, 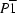

*a* = 9.5286 (2) Å
*b* = 10.1734 (2) Å
*c* = 13.2876 (3) Å
α = 72.831 (3)°
β = 75.741 (3)°
γ = 67.172 (2)°
*V* = 1121.51 (5) Å^3^
*Z* = 1
Mo *K*α radiation
μ = 1.02 mm^−1^
*T* = 100 K
0.37 × 0.26 × 0.10 mm

#### Data collection

Bruker Kappa APEXII CCD area-detector diffractometer
Absorption correction: multi-scan (*SADABS*; Bruker, 2005 ▶) *T*_min_ = 0.734, *T*_max_ = 0.901
20025 measured reflections
5581 independent reflections
5250 reflections with *I* > 2σ(*I*)
*R*_int_ = 0.023

#### Refinement

*R*[*F* ^2^ > 2σ(*F* ^2^)] = 0.024
*wR*(*F* ^2^) = 0.061
*S* = 1.08
5581 reflections
324 parameters
1 restraint
H atoms treated by a mixture of independent and constrained refinement
Δρ_max_ = 1.77 e Å^−3^
Δρ_min_ = −0.48 e Å^−3^

### 

Data collection: *APEX2* (Bruker, 2007 ▶); cell refinement: *SAINT* (Bruker, 2007 ▶); data reduction: *SAINT*; program(s) used to solve structure: *SHELXS97* (Sheldrick, 2008 ▶); program(s) used to refine structure: *SHELXL97* (Sheldrick, 2008 ▶); molecular graphics: *Mercury* (Macrae *et al.*, 2006 ▶); software used to prepare material for publication: *WinGX* (Farrugia, 1999 ▶) and *PLATON* (Spek, 2009 ▶).

## Supplementary Material

Crystal structure: contains datablocks I, global. DOI: 10.1107/S1600536810046258/xu5083sup1.cif

Structure factors: contains datablocks I. DOI: 10.1107/S1600536810046258/xu5083Isup2.hkl

Additional supplementary materials:  crystallographic information; 3D view; checkCIF report

## Figures and Tables

**Table 1:** Selected bond lengths (Å)

Cd1—N1	2.3265 (15)
Cd1—O1	2.3170 (14)
Cd1—O2	2.3844 (13)
Cd1—O3	2.5099 (15)
Cd1—O4	2.3185 (14)
Cd1—O4^i^	2.5625 (13)
Cd1—O6	2.3152 (14)

Symmetry code: (i) 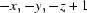.

**Table 2:** Hydrogen-bond geometry (Å, °)

*D*—H⋯*A*	*D*—H	H⋯*A*	*D*⋯*A*	*D*—H⋯*A*
N2—H2*A*⋯O1^i^	0.83 (3)	2.13 (3)	2.921 (2)	160 (2)
N2—H2*B*⋯O3^ii^	0.86 (3)	2.05 (3)	2.901 (3)	170 (3)
O6—H61⋯O5^iii^	0.79 (4)	1.91 (4)	2.692 (2)	167 (3)
O6—H62⋯O2^iv^	0.81 (4)	1.94 (4)	2.743 (2)	179 (3)
C11—H11⋯O2^i^	0.93	2.39	3.299 (2)	165
C17—H17⋯O1^i^	0.93	2.36	3.216 (3)	153
C21—H21⋯O2^iv^	0.93	2.45	3.252 (3)	145
C19—H19⋯*Cg*3^v^	0.93	2.74	3.537 (2)	144

Symmetry codes: (i) 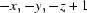; (ii) 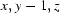; (iii) 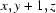; (iv) 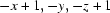; (v) 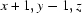.

**Fig. 1.**
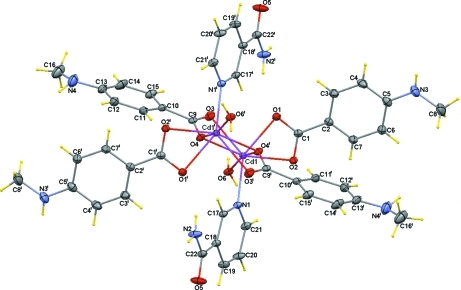
The molecular structure of the title compound with the atom-numbering scheme. Displacement ellipsoids are drawn at the 40% probability level. Primed atoms are generated by the symmetry operators: (') - x, - y, 1 - z.

## supplementary crystallographic information

### Comment

As a part of our ongoing investigation on transition metal complexes of nicotinamide (NA), one form of niacin (Krishnamachari, 1974), and/or the nicotinic acid derivative *N*,*N*-diethylnicotinamide (DENA), an important respiratory stimulant (Bigoli *et al.*, 1972), the title compound was synthesized and its crystal structure is reported herein.

The title compound, (I), consists of dimeric units located around a crystallographic symmetry centre and made up of two Cd cations, four 4-methylaminobenzoate (PMAB) anions, two nicotinamide (NA) ligands and two water molecules (Fig. 1). Each Cd(II) unit is chelated by the carboxylate O atoms of the two PMAB anions, and the two monomeric units are bridged through the two oxygen atoms of the two carboxylate groups about an inversion center. The coordination number of each Cd^II^ atom is seven. The Cd1···Cd1^i^ distance is 3.8204 (14) Å and O4-Cd1-O4^i^ angle is 77.12 (5)° (symmetry code: (i) -x, -y, 1 - z).

The average Cd-O bond length (Table 1) is 2.4013 (14) Å, and the Cd atom is displaced out of the least-squares planes of the carboxylate groups (O1/C1/O2) and (O3/C9/O4) by 0.4159 (1) and 0.4085 (1) Å, respectively. In (I), the O1-Cd1-O2 and O3-Cd1-O4 angles are 55.71 (5) and 117.52 (4) °, respectively. The corresponding O-M-O (where M is a metal) angles are 55.96 (4)° and 53.78 (4)° in [Cd_2_(DMAB)_4_(NA)_2_(H_2_O)_2_] (Hökelek *et al.*, 2010), 52.91 (4)° and 53.96 (4)° in [Cd(FB)_2_(INA)_2_(H_2_O)].H_2_O (Hökelek *et al.*, 2009*a*), 60.70 (4)° in [Co(DMAB)_2_(INA)(H_2_O)_2_] (Hökelek *et al.*, 2009*b*), 58.45 (9)° in [Mn(DMAB)_2_(INA)(H_2_O)_2_] (Hökelek *et al.*, 2009*c*), 60.03 (6)° in [Zn(MAB)_2_(INA)_2_].H_2_O (Hökelek *et al.*, 2009*d*), 58.3 (3)° in [Zn_2_(DENA)_2_(HB)_4_].2H_2_O (Hökelek & Necefoğlu, 1996) [where NA, INA, DENA, HB, FB, MAB and DMAB are nicotinamide, isonicotinamide, *N*,*N*-diethylnicotinamide, 4-hydroxybenzoate, 4-formylbenzoate, 4-methylaminobenzoate and 4-dimethylaminobenzoate, respectively] and 55.2 (1)° in [Cu(Asp)_2_(py)_2_] (where Asp is acetylsalicylate and py is pyridine) (Greenaway *et al.*, 1984).

The dihedral angles between the planar carboxylate groups and the adjacent benzene rings A (C2-C7) and B (C10-C15) are 9.86 (16) and 11.74 (11) °, respectively, while those between rings A, B, C (N1/C17-C21), D (Cd1/O1/O2/C1) and E (Cd1/O3/O4/C9) are A/B = 88.35 (6), A/C = 62.90 (7), B/C = 73.43 (6) and D/E = 63.44 (5)°.

In the crystal structure, intermolecular O-H···O, N-H···O and C-H···O hydrogen bonds (Table 2) link the molecules into a three-dimensional network, in which they may be effective in the stabilization of the structure. The π–π contact between the pyridine rings, Cg3—Cg3^i^ [symmetry code: (i) 1 - x, -1 - y, 1 - z, where Cg3 is the centroid of the ring C (N3/C17-C21)] may further stabilize the structure, with centroid-centroid distance of 3.965 (1) Å. There also exists a weak C-H···π interaction (Table 2).

### Experimental

The title compound was prepared by the reaction of 3CdSO_4_.H_2_O (1.08 g, 5 mmol) in H_2_O (30 ml) and NA (1.22 g, 10 mmol) in H_2_O (20 ml) with sodium 4-(methylamino)benzoate (1.73 g, 10 mmol) in H_2_O (150 ml). The mixture was filtered and set aside to crystallize at ambient temperature for one week, giving colorless single crystals.

### Refinement

Atoms H3A, H4A (for NH), H2A, H2B (for NH_2_) and H61, H62 (for H_2_O) were located in a difference Fourier map and refined isotropically. The remaining H atoms were positioned geometrically with C—H = 0.93 and 0.96 Å for aromatic and methyl H atoms and constrained to ride on their parent atoms, with *U*_iso_(H) = *xU*_eq_(C), where *x* = 1.5 for methyl H and *x* = 1.2 for aromatic H atoms.

### Crystal data

**Table:**

[Cd_2_(C_8_H_8_NO_2_)_4_(C_6_H_6_N_2_O)_2_(H_2_O)_2_]	*Z* = 1
*M**_r_* = 1105.72	*F*(000) = 560
Triclinic, *P*1	*D*_x_ = 1.637 Mg m^−^^3^
Hall symbol: -P 1	Mo *K*α radiation, λ = 0.71073 Å
*a* = 9.5286 (2) Å	Cell parameters from 9941 reflections
*b* = 10.1734 (2) Å	θ = 2.4–28.4°
*c* = 13.2876 (3) Å	µ = 1.02 mm^−^^1^
α = 72.831 (3)°	*T* = 100 K
β = 75.741 (3)°	Block, colorless
γ = 67.172 (2)°	0.37 × 0.26 × 0.10 mm
*V* = 1121.51 (5) Å^3^	

### Data collection

**Table:**

Bruker Kappa APEXII CCD area-detector diffractometer	5581 independent reflections
Radiation source: fine-focus sealed tube	5250 reflections with *I* > 2σ(*I*)
graphite	*R*_int_ = 0.023
φ and ω scans	θ_max_ = 28.4°, θ_min_ = 1.6°
Absorption correction: multi-scan (*SADABS*; Bruker, 2005)	*h* = −12→12
*T*_min_ = 0.734, *T*_max_ = 0.901	*k* = −13→13
20025 measured reflections	*l* = −17→16

### Refinement

**Table:**

Refinement on *F*^2^	Primary atom site location: structure-invariant direct methods
Least-squares matrix: full	Secondary atom site location: difference Fourier map
*R*[*F*^2^ > 2σ(*F*^2^)] = 0.024	Hydrogen site location: inferred from neighbouring sites
*wR*(*F*^2^) = 0.061	H atoms treated by a mixture of independent and constrained refinement
*S* = 1.08	*w* = 1/[σ^2^(*F*_o_^2^) + (0.0294*P*)^2^ + 0.9733*P*] where *P* = (*F*_o_^2^ + 2*F*_c_^2^)/3
5581 reflections	(Δ/σ)_max_ < 0.001
324 parameters	Δρ_max_ = 1.77 e Å^−^^3^
1 restraint	Δρ_min_ = −0.48 e Å^−^^3^

### Special details

**Table:**

Geometry. All esds (except the esd in the dihedral angle between two l.s. planes) are estimated using the full covariance matrix. The cell esds are taken into account individually in the estimation of esds in distances, angles and torsion angles; correlations between esds in cell parameters are only used when they are defined by crystal symmetry. An approximate (isotropic) treatment of cell esds is used for estimating esds involving l.s. planes.
Refinement. Refinement of F^2^ against ALL reflections. The weighted R-factor wR and goodness of fit S are based on F^2^, conventional R-factors R are based on F, with F set to zero for negative F^2^. The threshold expression of F^2^ > 2sigma(F^2^) is used only for calculating R-factors(gt) etc. and is not relevant to the choice of reflections for refinement. R-factors based on F^2^ are statistically about twice as large as those based on F, and R- factors based on ALL data will be even larger.

### Fractional atomic coordinates and isotropic or equivalent isotropic displacement parameters (Å^2^)

**Table:**

	*x*	*y*	*z*	*U*_iso_*/*U*_eq_	
Cd1	0.183285 (14)	0.043205 (13)	0.484176 (10)	0.01391 (5)	
O1	0.08486 (15)	0.16573 (15)	0.62175 (12)	0.0210 (3)	
O2	0.32071 (15)	0.00805 (14)	0.62305 (11)	0.0175 (3)	
O3	0.04603 (16)	0.29436 (15)	0.38267 (12)	0.0234 (3)	
O4	−0.01778 (15)	0.10432 (14)	0.39110 (11)	0.0175 (3)	
O5	0.37181 (18)	−0.60649 (17)	0.33609 (16)	0.0357 (4)	
O6	0.37108 (16)	0.12104 (15)	0.36256 (12)	0.0188 (3)	
H61	0.357 (3)	0.205 (4)	0.357 (2)	0.039 (8)*	
H62	0.462 (4)	0.084 (3)	0.366 (2)	0.042 (8)*	
N1	0.32666 (17)	−0.18369 (16)	0.44351 (13)	0.0157 (3)	
N2	0.1327 (2)	−0.4634 (2)	0.38591 (18)	0.0281 (4)	
H2A	0.071 (3)	−0.385 (3)	0.400 (2)	0.037 (7)*	
H2B	0.099 (3)	−0.528 (3)	0.380 (2)	0.039 (8)*	
N3	0.1429 (3)	0.0766 (2)	1.10668 (16)	0.0335 (4)	
H3A	0.051 (4)	0.113 (4)	1.137 (3)	0.053 (9)*	
N4	−0.3405 (3)	0.5412 (3)	−0.00800 (18)	0.0413 (5)	
H4A	−0.345 (5)	0.491 (5)	−0.048 (3)	0.100 (17)*	
C1	0.2004 (2)	0.09189 (19)	0.66971 (15)	0.0155 (3)	
C2	0.1916 (2)	0.0959 (2)	0.78140 (15)	0.0170 (3)	
C3	0.0524 (2)	0.1725 (2)	0.83657 (17)	0.0232 (4)	
H3	−0.0311	0.2287	0.8004	0.028*	
C4	0.0371 (2)	0.1661 (2)	0.94354 (18)	0.0276 (4)	
H4	−0.0565	0.2179	0.9785	0.033*	
C5	0.1613 (2)	0.0820 (2)	1.00047 (16)	0.0240 (4)	
C6	0.3012 (2)	0.0073 (2)	0.94509 (17)	0.0251 (4)	
H6	0.3854	−0.0478	0.9808	0.030*	
C7	0.3151 (2)	0.0148 (2)	0.83732 (16)	0.0227 (4)	
H7	0.4091	−0.0354	0.8017	0.027*	
C8	0.2541 (4)	−0.0243 (3)	1.1746 (2)	0.0446 (6)	
H8A	0.2118	−0.0199	1.2474	0.067*	
H8B	0.3456	0.0014	1.1554	0.067*	
H8C	0.2792	−0.1218	1.1661	0.067*	
C9	−0.0265 (2)	0.23868 (19)	0.35120 (15)	0.0163 (3)	
C10	−0.1183 (2)	0.32451 (19)	0.26270 (15)	0.0171 (3)	
C11	−0.2098 (2)	0.2690 (2)	0.23138 (16)	0.0207 (4)	
H11	−0.2212	0.1806	0.2710	0.025*	
C12	−0.2835 (2)	0.3414 (2)	0.14353 (18)	0.0265 (4)	
H12	−0.3439	0.3015	0.1251	0.032*	
C13	−0.2687 (2)	0.4745 (2)	0.08146 (18)	0.0288 (5)	
C14	−0.1827 (3)	0.5343 (2)	0.1148 (2)	0.0324 (5)	
H14	−0.1749	0.6246	0.0769	0.039*	
C15	−0.1087 (2)	0.4601 (2)	0.20394 (18)	0.0248 (4)	
H15	−0.0520	0.5016	0.2246	0.030*	
C16	−0.3123 (3)	0.6668 (3)	−0.0834 (2)	0.0487 (7)	
H16A	−0.3610	0.6892	−0.1446	0.073*	
H16B	−0.2034	0.6454	−0.1052	0.073*	
H16C	−0.3538	0.7491	−0.0506	0.073*	
C17	0.2634 (2)	−0.26755 (19)	0.42175 (15)	0.0157 (3)	
H17	0.1566	−0.2411	0.4335	0.019*	
C18	0.3509 (2)	−0.39229 (19)	0.38237 (15)	0.0164 (3)	
C19	0.5101 (2)	−0.4288 (2)	0.36180 (18)	0.0221 (4)	
H19	0.5715	−0.5092	0.3328	0.027*	
C20	0.5760 (2)	−0.3436 (2)	0.38513 (19)	0.0244 (4)	
H20	0.6823	−0.3663	0.3725	0.029*	
C21	0.4810 (2)	−0.2241 (2)	0.42749 (17)	0.0193 (4)	
H21	0.5258	−0.1694	0.4456	0.023*	
C22	0.2834 (2)	−0.4945 (2)	0.36559 (16)	0.0188 (4)	

### Atomic displacement parameters (Å^2^)

**Table:**

	*U*^11^	*U*^22^	*U*^33^	*U*^12^	*U*^13^	*U*^23^
Cd1	0.01123 (7)	0.01258 (7)	0.01849 (7)	−0.00263 (5)	−0.00261 (5)	−0.00593 (5)
O1	0.0179 (6)	0.0192 (6)	0.0262 (7)	−0.0015 (5)	−0.0074 (5)	−0.0088 (6)
O2	0.0140 (6)	0.0195 (6)	0.0188 (6)	−0.0045 (5)	−0.0017 (5)	−0.0063 (5)
O3	0.0197 (7)	0.0205 (7)	0.0329 (8)	−0.0079 (5)	−0.0083 (6)	−0.0050 (6)
O4	0.0160 (6)	0.0144 (6)	0.0197 (7)	−0.0047 (5)	−0.0032 (5)	−0.0003 (5)
O5	0.0222 (7)	0.0254 (8)	0.0654 (12)	−0.0095 (6)	0.0063 (7)	−0.0276 (8)
O6	0.0144 (6)	0.0140 (6)	0.0246 (7)	−0.0028 (5)	−0.0022 (5)	−0.0031 (5)
N1	0.0139 (7)	0.0135 (7)	0.0195 (8)	−0.0047 (6)	−0.0023 (6)	−0.0035 (6)
N2	0.0165 (8)	0.0182 (8)	0.0551 (13)	−0.0045 (7)	−0.0054 (8)	−0.0178 (8)
N3	0.0388 (11)	0.0394 (11)	0.0193 (9)	−0.0110 (9)	0.0017 (8)	−0.0103 (8)
N4	0.0413 (12)	0.0427 (13)	0.0295 (11)	−0.0085 (10)	−0.0138 (9)	0.0059 (10)
C1	0.0152 (8)	0.0129 (7)	0.0199 (9)	−0.0072 (6)	−0.0012 (7)	−0.0037 (7)
C2	0.0170 (8)	0.0171 (8)	0.0187 (9)	−0.0080 (7)	−0.0005 (7)	−0.0054 (7)
C3	0.0185 (9)	0.0243 (9)	0.0265 (10)	−0.0051 (7)	−0.0019 (8)	−0.0092 (8)
C4	0.0214 (10)	0.0338 (11)	0.0274 (11)	−0.0078 (9)	0.0038 (8)	−0.0146 (9)
C5	0.0299 (10)	0.0247 (9)	0.0199 (10)	−0.0138 (8)	0.0017 (8)	−0.0070 (8)
C6	0.0236 (10)	0.0280 (10)	0.0213 (10)	−0.0049 (8)	−0.0052 (8)	−0.0060 (8)
C7	0.0184 (9)	0.0268 (10)	0.0211 (10)	−0.0043 (8)	−0.0016 (7)	−0.0085 (8)
C8	0.0619 (18)	0.0446 (15)	0.0201 (11)	−0.0107 (13)	−0.0055 (11)	−0.0079 (10)
C9	0.0109 (8)	0.0155 (8)	0.0193 (9)	−0.0021 (6)	0.0002 (6)	−0.0045 (7)
C10	0.0143 (8)	0.0138 (8)	0.0188 (9)	−0.0019 (6)	−0.0010 (7)	−0.0024 (7)
C11	0.0209 (9)	0.0163 (8)	0.0233 (10)	−0.0036 (7)	−0.0057 (7)	−0.0037 (7)
C12	0.0258 (10)	0.0246 (10)	0.0279 (11)	−0.0039 (8)	−0.0096 (8)	−0.0061 (8)
C13	0.0226 (10)	0.0263 (10)	0.0242 (10)	0.0016 (8)	−0.0048 (8)	0.0011 (8)
C14	0.0267 (11)	0.0198 (10)	0.0364 (13)	−0.0050 (8)	−0.0034 (9)	0.0093 (9)
C15	0.0200 (9)	0.0173 (9)	0.0328 (11)	−0.0068 (7)	−0.0034 (8)	0.0007 (8)
C16	0.0348 (14)	0.0494 (16)	0.0388 (15)	−0.0064 (12)	−0.0058 (11)	0.0129 (12)
C17	0.0125 (8)	0.0144 (8)	0.0200 (9)	−0.0045 (6)	−0.0021 (6)	−0.0039 (7)
C18	0.0157 (8)	0.0123 (8)	0.0210 (9)	−0.0056 (6)	−0.0021 (7)	−0.0029 (7)
C19	0.0161 (9)	0.0146 (8)	0.0334 (11)	−0.0036 (7)	0.0009 (8)	−0.0081 (8)
C20	0.0118 (8)	0.0186 (9)	0.0414 (12)	−0.0052 (7)	0.0001 (8)	−0.0079 (8)
C21	0.0152 (8)	0.0142 (8)	0.0292 (10)	−0.0061 (7)	−0.0047 (7)	−0.0035 (7)
C22	0.0185 (9)	0.0140 (8)	0.0245 (10)	−0.0058 (7)	−0.0035 (7)	−0.0046 (7)

### Geometric parameters (Å, °)

**Table:**

Cd1—N1	2.3265 (15)	C6—H6	0.9300
Cd1—O1	2.3170 (14)	C7—C6	1.387 (3)
Cd1—O2	2.3844 (13)	C7—H7	0.9300
Cd1—O3	2.5099 (15)	C8—H8A	0.9600
Cd1—O4	2.3185 (14)	C8—H8B	0.9600
Cd1—O4^i^	2.5625 (13)	C8—H8C	0.9600
Cd1—O6	2.3152 (14)	C9—C10	1.489 (3)
Cd1—C1	2.7077 (18)	C10—C11	1.397 (3)
O1—C1	1.267 (2)	C10—C15	1.395 (3)
O2—C1	1.276 (2)	C11—C12	1.376 (3)
O3—C9	1.249 (2)	C11—H11	0.9300
O4—C9	1.289 (2)	C12—C13	1.402 (3)
O5—C22	1.232 (2)	C12—H12	0.9300
O6—H61	0.80 (3)	C13—N4	1.381 (3)
O6—H62	0.81 (3)	C13—C14	1.401 (4)
N1—C17	1.344 (2)	C14—H14	0.9300
N1—C21	1.343 (2)	C15—C14	1.393 (3)
N2—C22	1.319 (3)	C15—H15	0.9300
N2—H2A	0.83 (3)	C16—N4	1.444 (3)
N2—H2B	0.86 (3)	C16—H16A	0.9600
N3—C5	1.366 (3)	C16—H16B	0.9600
N3—C8	1.439 (4)	C16—H16C	0.9600
N3—H3A	0.86 (3)	C17—C18	1.391 (2)
N4—H4A	0.86 (4)	C17—H17	0.9300
C2—C1	1.478 (3)	C19—C18	1.390 (3)
C2—C3	1.400 (3)	C19—C20	1.385 (3)
C2—C7	1.390 (3)	C19—H19	0.9300
C3—H3	0.9300	C20—H20	0.9300
C4—C3	1.377 (3)	C21—C20	1.383 (3)
C4—H4	0.9300	C21—H21	0.9300
C5—C4	1.407 (3)	C22—C18	1.507 (2)
C5—C6	1.399 (3)		
			
O1—Cd1—O2	55.71 (5)	C3—C4—H4	119.6
O1—Cd1—O3	80.21 (5)	C5—C4—H4	119.6
O1—Cd1—O4	106.64 (5)	N3—C5—C4	119.6 (2)
O1—Cd1—O4^i^	79.05 (5)	N3—C5—C6	122.2 (2)
O1—Cd1—N1	143.81 (5)	C6—C5—C4	118.18 (19)
O1—Cd1—C1	27.83 (5)	C5—C6—H6	119.8
O2—Cd1—O3	121.55 (5)	C7—C6—C5	120.5 (2)
O2—Cd1—O4^i^	91.77 (4)	C7—C6—H6	119.8
O2—Cd1—C1	28.12 (5)	C2—C7—H7	119.3
O3—Cd1—O4^i^	117.52 (4)	C6—C7—C2	121.35 (18)
O3—Cd1—C1	103.17 (5)	C6—C7—H7	119.3
O4—Cd1—O2	161.16 (5)	N3—C8—H8A	109.5
O4—Cd1—O3	54.22 (4)	N3—C8—H8B	109.5
O4—Cd1—O4^i^	77.12 (5)	N3—C8—H8C	109.5
O4—Cd1—N1	98.06 (5)	H8A—C8—H8B	109.5
O4—Cd1—C1	133.53 (5)	H8A—C8—H8C	109.5
O4^i^—Cd1—C1	82.17 (5)	H8B—C8—H8C	109.5
O6—Cd1—O1	112.54 (5)	O3—C9—O4	120.79 (17)
O6—Cd1—O2	88.69 (5)	O3—C9—C10	120.31 (17)
O6—Cd1—O3	73.74 (5)	O4—C9—C10	118.82 (16)
O6—Cd1—O4	105.81 (5)	C11—C10—C9	121.45 (17)
O6—Cd1—O4^i^	165.88 (5)	C15—C10—C9	120.91 (18)
O6—Cd1—N1	84.67 (5)	C15—C10—C11	117.54 (18)
O6—Cd1—C1	104.26 (5)	C10—C11—H11	119.1
N1—Cd1—O2	95.16 (5)	C12—C11—C10	121.82 (19)
N1—Cd1—O3	135.97 (5)	C12—C11—H11	119.1
N1—Cd1—O4^i^	81.23 (5)	C11—C12—C13	120.8 (2)
N1—Cd1—C1	119.34 (6)	C11—C12—H12	119.6
C1—O1—Cd1	93.51 (11)	C13—C12—H12	119.6
C1—O2—Cd1	90.17 (11)	N4—C13—C12	119.0 (2)
C9—O3—Cd1	88.13 (11)	N4—C13—C14	123.2 (2)
Cd1—O4—Cd1^i^	102.88 (5)	C14—C13—C12	117.8 (2)
C9—O4—Cd1	95.93 (11)	C13—C14—H14	119.6
C9—O4—Cd1^i^	139.46 (11)	C15—C14—C13	120.8 (2)
Cd1—O6—H61	113 (2)	C15—C14—H14	119.6
Cd1—O6—H62	124 (2)	C10—C15—H15	119.5
H61—O6—H62	102 (3)	C14—C15—C10	121.1 (2)
C17—N1—Cd1	123.06 (12)	C14—C15—H15	119.5
C21—N1—Cd1	118.29 (12)	N4—C16—H16A	109.5
C21—N1—C17	118.08 (15)	N4—C16—H16B	109.5
C22—N2—H2A	123 (2)	N4—C16—H16C	109.5
C22—N2—H2B	116.2 (19)	H16A—C16—H16B	109.5
H2A—N2—H2B	120 (3)	H16A—C16—H16C	109.5
C5—N3—C8	123.2 (2)	H16B—C16—H16C	109.5
C5—N3—H3A	117 (2)	N1—C17—C18	122.70 (16)
C8—N3—H3A	117 (2)	N1—C17—H17	118.6
C13—N4—C16	121.7 (2)	C18—C17—H17	118.6
C13—N4—H4A	122 (3)	C17—C18—C22	123.63 (16)
C16—N4—H4A	103 (3)	C19—C18—C17	118.41 (17)
O1—C1—Cd1	58.66 (9)	C19—C18—C22	117.89 (16)
O1—C1—O2	119.58 (17)	C18—C19—H19	120.5
O1—C1—C2	119.89 (16)	C20—C19—C18	119.07 (17)
O2—C1—Cd1	61.71 (10)	C20—C19—H19	120.5
O2—C1—C2	120.41 (17)	C19—C20—H20	120.6
C2—C1—Cd1	167.59 (12)	C21—C20—C19	118.87 (17)
C3—C2—C1	120.01 (17)	C21—C20—H20	120.6
C7—C2—C1	121.66 (17)	N1—C21—C20	122.78 (17)
C7—C2—C3	118.10 (18)	N1—C21—H21	118.6
C2—C3—H3	119.4	C20—C21—H21	118.6
C4—C3—C2	121.1 (2)	O5—C22—N2	122.37 (18)
C4—C3—H3	119.4	O5—C22—C18	118.50 (17)
C3—C4—C5	120.76 (19)	N2—C22—C18	119.10 (16)
			
O2—Cd1—O1—C1	−5.82 (10)	O6—Cd1—C1—C2	−161.5 (6)
O3—Cd1—O1—C1	−145.30 (11)	N1—Cd1—C1—O1	−156.66 (10)
O4—Cd1—O1—C1	166.82 (10)	N1—Cd1—C1—O2	33.58 (12)
O4^i^—Cd1—O1—C1	93.97 (11)	N1—Cd1—C1—C2	−69.7 (6)
O6—Cd1—O1—C1	−77.59 (11)	Cd1—O1—C1—O2	10.37 (17)
N1—Cd1—O1—C1	35.80 (15)	Cd1—O1—C1—C2	−165.67 (14)
O1—Cd1—O2—C1	5.76 (10)	Cd1—O2—C1—O1	−10.05 (16)
O3—Cd1—O2—C1	54.46 (11)	Cd1—O2—C1—C2	165.96 (14)
O4—Cd1—O2—C1	−16.57 (19)	Cd1—O3—C9—O4	9.37 (17)
O4^i^—Cd1—O2—C1	−69.69 (10)	Cd1—O3—C9—C10	−167.39 (15)
O6—Cd1—O2—C1	124.42 (10)	Cd1—O4—C9—O3	−10.20 (18)
N1—Cd1—O2—C1	−151.05 (10)	Cd1^i^—O4—C9—O3	107.6 (2)
O1—Cd1—O3—C9	−124.35 (11)	Cd1—O4—C9—C10	166.61 (13)
O2—Cd1—O3—C9	−163.39 (10)	Cd1^i^—O4—C9—C10	−75.6 (2)
O4—Cd1—O3—C9	−5.50 (10)	Cd1—N1—C17—C18	170.27 (14)
O4^i^—Cd1—O3—C9	−52.25 (12)	C21—N1—C17—C18	−0.8 (3)
O6—Cd1—O3—C9	118.55 (11)	Cd1—N1—C21—C20	−168.42 (16)
N1—Cd1—O3—C9	54.71 (13)	C17—N1—C21—C20	3.1 (3)
C1—Cd1—O3—C9	−140.19 (11)	C8—N3—C5—C4	169.5 (2)
O1—Cd1—O4—Cd1^i^	−74.23 (6)	C8—N3—C5—C6	−11.1 (4)
O1—Cd1—O4—C9	69.63 (11)	C3—C2—C1—Cd1	−75.0 (6)
O2—Cd1—O4—Cd1^i^	−55.11 (15)	C3—C2—C1—O1	4.7 (3)
O2—Cd1—O4—C9	88.75 (17)	C3—C2—C1—O2	−171.30 (17)
O3—Cd1—O4—Cd1^i^	−138.50 (7)	C7—C2—C1—Cd1	99.4 (6)
O3—Cd1—O4—C9	5.36 (10)	C7—C2—C1—O1	179.06 (17)
O4^i^—Cd1—O4—Cd1^i^	0.0	C7—C2—C1—O2	3.1 (3)
O4^i^—Cd1—O4—C9	143.86 (12)	C1—C2—C3—C4	173.52 (18)
O6—Cd1—O4—Cd1^i^	165.74 (5)	C7—C2—C3—C4	−1.0 (3)
O6—Cd1—O4—C9	−50.40 (11)	C1—C2—C7—C6	−173.35 (18)
N1—Cd1—O4—Cd1^i^	79.03 (5)	C3—C2—C7—C6	1.1 (3)
N1—Cd1—O4—C9	−137.11 (11)	C5—C4—C3—C2	0.0 (3)
C1—Cd1—O4—Cd1^i^	−65.79 (7)	N3—C5—C4—C3	−179.5 (2)
C1—Cd1—O4—C9	78.07 (12)	C6—C5—C4—C3	1.0 (3)
O1—Cd1—N1—C17	105.36 (15)	N3—C5—C6—C7	179.6 (2)
O1—Cd1—N1—C21	−83.55 (16)	C4—C5—C6—C7	−1.0 (3)
O2—Cd1—N1—C17	138.80 (14)	C2—C7—C6—C5	−0.1 (3)
O2—Cd1—N1—C21	−50.11 (15)	O3—C9—C10—C11	−175.08 (17)
O3—Cd1—N1—C17	−73.07 (16)	O3—C9—C10—C15	8.7 (3)
O3—Cd1—N1—C21	98.02 (15)	O4—C9—C10—C11	8.1 (3)
O4—Cd1—N1—C17	−27.75 (15)	O4—C9—C10—C15	−168.09 (17)
O4^i^—Cd1—N1—C17	47.80 (14)	C9—C10—C11—C12	−173.83 (18)
O4—Cd1—N1—C21	143.34 (14)	C15—C10—C11—C12	2.5 (3)
O4^i^—Cd1—N1—C21	−141.11 (15)	C9—C10—C15—C14	173.80 (19)
O6—Cd1—N1—C17	−133.01 (15)	C11—C10—C15—C14	−2.5 (3)
O6—Cd1—N1—C21	38.08 (14)	C10—C11—C12—C13	0.3 (3)
C1—Cd1—N1—C17	123.62 (14)	C11—C12—C13—N4	177.9 (2)
C1—Cd1—N1—C21	−65.29 (15)	C11—C12—C13—C14	−3.0 (3)
O1—Cd1—C1—O2	−169.76 (17)	C12—C13—N4—C16	−171.1 (2)
O1—Cd1—C1—C2	87.0 (6)	C14—C13—N4—C16	9.9 (4)
O2—Cd1—C1—O1	169.76 (17)	N4—C13—C14—C15	−178.1 (2)
O2—Cd1—C1—C2	−103.2 (6)	C12—C13—C14—C15	2.9 (3)
O3—Cd1—C1—O1	35.18 (11)	C10—C15—C14—C13	−0.1 (3)
O3—Cd1—C1—O2	−134.59 (10)	N1—C17—C18—C19	−2.0 (3)
O3—Cd1—C1—C2	122.2 (6)	N1—C17—C18—C22	174.98 (18)
O4—Cd1—C1—O1	−17.53 (13)	C20—C19—C18—C17	2.6 (3)
O4^i^—Cd1—C1—O1	−81.36 (11)	C20—C19—C18—C22	−174.56 (19)
O4—Cd1—C1—O2	172.70 (9)	C18—C19—C20—C21	−0.5 (3)
O4^i^—Cd1—C1—O2	108.88 (10)	N1—C21—C20—C19	−2.5 (3)
O4—Cd1—C1—C2	69.5 (6)	O5—C22—C18—C17	−175.9 (2)
O4^i^—Cd1—C1—C2	5.6 (6)	O5—C22—C18—C19	1.1 (3)
O6—Cd1—C1—O1	111.46 (11)	N2—C22—C18—C17	2.1 (3)
O6—Cd1—C1—O2	−58.31 (11)	N2—C22—C18—C19	179.1 (2)

Symmetry codes: (i) −*x*, −*y*, −*z*+1.

### Hydrogen-bond geometry (Å, °)

**Table:**

*D*—H···*A*	*D*—H	H···*A*	*D*···*A*	*D*—H···*A*
N2—H2A···O1^i^	0.83 (3)	2.13 (3)	2.921 (2)	160 (2)
N2—H2B···O3^ii^	0.86 (3)	2.05 (3)	2.901 (3)	170 (3)
O6—H61···O5^iii^	0.79 (4)	1.91 (4)	2.692 (2)	167 (3)
O6—H62···O2^iv^	0.81 (4)	1.94 (4)	2.743 (2)	179 (3)
C11—H11···O2^i^	0.93	2.39	3.299 (2)	165
C17—H17···O1^i^	0.93	2.36	3.216 (3)	153
C21—H21···O2^iv^	0.93	2.45	3.252 (3)	145
C19—H19···Cg3^v^	0.93	2.74	3.537 (2)	144

Symmetry codes: (i) −*x*, −*y*, −*z*+1; (ii) *x*, *y*−1, *z*; (iii) *x*, *y*+1, *z*; (iv) −*x*+1, −*y*, −*z*+1; (v) *x*+1, *y*−1, *z*.
